# Collagen-I influences the post-translational regulation, binding partners and role of Annexin A2 in breast cancer progression

**DOI:** 10.3389/fonc.2023.1270436

**Published:** 2023-10-24

**Authors:** Amira F. Mahdi, Joanne Nolan, Ruth Í. O’Connor, Aoife J. Lowery, Joanna M. Allardyce, Patrick A. Kiely, Kieran McGourty

**Affiliations:** ^1^ School of Medicine, University of Limerick, Limerick, Ireland; ^2^ Health Research Institute, University of Limerick, Limerick, Ireland; ^3^ Lambe Institute for Translational Research, University of Galway, Galway, Ireland; ^4^ School of Allied Health, University of Limerick, Limerick, Ireland; ^5^ Science Foundation Ireland Research Centre in Pharmaceuticals (SSPC), University of Limerick, Limerick, Ireland; ^6^ Department of Chemical Sciences, Bernal Institute, University of Limerick, Limerick, Ireland

**Keywords:** breast cancer, Annexin A2, metastasis, collagen-I, extracellular matrix, tumor microenvironment

## Abstract

**Introduction:**

The extracellular matrix (ECM) has been heavily implicated in the development and progression of cancer. We have previously shown that Annexin A2 is integral in the migration and invasion of breast cancer cells and in the clinical progression of ER-negative breast cancer, processes which are highly influenced by the surrounding tumor microenvironment and ECM.

**Methods:**

We investigated how modulations of the ECM may affect the role of Annexin A2 in MDA-MB-231 breast cancer cells using western blotting, immunofluorescent confocal microscopy and immuno-precipitation mass spectrometry techniques.

**Results:**

We have shown that the presence of collagen-I, the main constituent of the ECM, increases the post-translational phosphorylation of Annexin A2 and subsequently causes the translocation of Annexin A2 to the extracellular surface. In the presence of collagen-I, we identified fibronectin as a novel interactor of Annexin A2, using mass spectrometry analysis. We then demonstrated that reducing Annexin A2 expression decreases the degradation of fibronectin by cancer cells and this effect on fibronectin turnover is increased according to collagen-I abundance.

**Discussion:**

Our results suggest that Annexin A2's role in promoting cancer progression is mediated by collagen-I and Annexin A2 maybe a therapeutic target in the bi-directional cross-talk between cancer cells and ECM remodeling that supports metastatic cancer progression.

## 1 Introduction

The study of cancer has evolved over decades to encompass not only malignant cells, but also those cells’ neighbors and surroundings, known as the tumor microenvironment (TME). Apart from stromal and immune cells, the principal component of the TME is the extracellular matrix (ECM). Comprised of a diverse array of proteoglycans and fibrillar proteins, the ECM can be thought of as a 3-dimenstional scaffold which gives structure and physical support to cells and tissues. A tangible paradigm shift has occurred in our understanding of the ECM, as we now understand it is not simply an inert bystander in homeostasis, but rather it is a dynamic and active component in maintaining appropriate cellular signaling pathways and tissue integrity (1). This dynamicity can be hijacked in the malignant process, wherein the normal levels of organization between tissue and stroma become disrupted as tumor cells aberrantly remodel, dysregulate and invade their microenvironment. An over-expression of ECM proteins and ECM remodeling enzymes are found in a variety of solid cancer types (2, 3) often leading to the creation of a desmoplastic TME. This is characterized by the increased deposition and cross-linking of fibrotic ECM proteins, such as collagens, in particular collagen Type I (collagen-I) (4, 5).

In human breast cancers, a desmoplastic or fibrotic reaction, triggered by altered deposition and remodeling of collagen-I, is often the first noticeable sign of breast cancer development and is a common characteristic of the disease with pathological consequence (6). Previous work by our lab has shown that the presence of collagen-I and fibronectin increases both the proliferation and migration of breast cancer cells (7). Collagen abundance and fibre density has been associated with breast tumor growth (8, 9), spread to the lymph nodes (10) and lung metastases (11). Furthermore, high mammographic breast density, a characteristic strongly associated with increased collagen abundance (12, 13), is linked to a 4–6 fold increase in breast cancer risk, to metastatic progression and to overall poor prognosis (13–17).

TME dysregulation is multifactorial and, in its essence, results from aberrant inside-out and outside-in signaling between cancer cells and their surroundings. One such mediator for this TME-cancer cell cross-talk is Annexin A2 due to its transient intra- to extra-cellular cycling and its role in ECM degradation. The translocation of Annexin A2 is regulated by its binding to S100A10 and by its post-translational modifications (18, 19). Phosphorylation of Annexin A2 at Tyr24 (aka Tyr23) by the Src family of kinases, promotes Annexin A2 expression on the cell surface (18, 20–23). Conversely, phosphorylation at Ser26 (aka Ser25) by Protein Kinase C (PKC) inhibits Annexin A2 surface expression by interrupting its binding to S100A10 (23–25). Once on the extracellular surface, Annexin A2 plays a role in ECM remodeling by acting as a co-receptor for plasminogen and tissue-type plasminogen activator (t-PA), thus promoting the localized activation of plasmin, a serine protease known to degrade ECM proteins such as fibronectin and laminin (26–29). Activated plasmin also functions to release sequestered growth factors in the TME and further activate matrix metalloproteases (MMPs), triggering a proteolytic cascade, which increases the invasive capacity of cancer cells (30–32). Despite these molecular insights and a number of links between Annexin A2 phosphorylation and cancer, there is scant information regarding the physiological regulation and implications of this in the context of the TME.

Our previous work revealed Annexin A2 to be a newly synthesized protein in the growth factor induced migration and invasion of breast cancer cells. Further to this, we showed that Annexin A2’s expression is required to support the proliferation and migration of cells *in vitro*. In patients, we found Annexin A2 expression to be higher in aggressive, ER negative subtypes of breast cancer and to be associated with a higher risk of metastatic progression (33). In addition to our findings, several other studies have linked Annexin A2 to cancer progression as reviewed in (19, 34, 35). Similarly, ECM dysregulation, including desmoplasia and increased collagen expression has been also linked to the metastatic process (36, 37). Consequently, we wanted to investigate the potential relationship between ECM dysregulation and Annexin A2 expression as both represent hallmarks of aggressive cancer progression (35, 38). Considering Annexin A2’s well documented role in the activation of ECM remodeling proteases, this led to our rational to investigate the relationship here as a potential explanation for our previous findings on Annexin A2’s role in metastatic processes and patient prognosis.

## 2 Materials and methods

### 2.1 Cell culture

Human breast cancer cell lines MDA-MB-231 were purchased from the ECACC culture collection (Sigma Aldrich, Wicklow, Ireland). Cell lines were routinely tested upon freezing and thawing for mycoplasma contamination using PCR. Cells were maintained at 37°C, 5% CO2 humidified incubator in DMEM-high glucose supplemented with 10% fetal bovine serum, 5% l-glutamine and 5% penicillin/streptomycin (all obtained from Sigma Aldrich).

### 2.2 Extracellular matrix coating of tissue culture surface

All matrix proteins were diluted in sterile 1 X PBS. Concentrations used were as follows: Collagen-I = 1.8 µg/cm^2^, fibronectin = 20µg/cm^2^. Following coating, plates were incubated at 37°C for 4 hours before the excess coating was aspirated off and cells plated onto the surface.

### 2.3 Cell lysis, SDS PAGE and western blotting

The cells were lysed in 1% NP-40 lysis buffer then quantified and denatured with SDS loading buffer and boiled for 5 min. Lysates were separated on 12% SDS acrylamide gels and subsequently transferred to nitrocellulose membrane. Membranes were blocked using 5% BSA for 1 h at room temperature then probed with corresponding primary antibodies at 4°C overnight. Dilutions of antibodies were as follows: Annexin A2 1:1000 (Abcam, Cambridge UK), Annexin A2 1:1000 (BD Bioscience), B-Actin 1:1000 (Sigma), phospo-Tyr24-Annexin A2 1:250 (Santa Cruz Biotechnology, Heidelberg, GE) GAPDH 1:1000 (Sigma) Na/K-ATPase 1:500 (Cell Signaling, Beverly, MA), Fibronectin 1:100, (Santa Cruz). IRdye700- or IRdye800-conjugated secondary antibodies, were then coupled to the primary antibody for 1 h at room temperature. Protein bands were detected using the Odyssey Sc (LI-COR, Cambridge, UK) and quantified using Image Studio 5.2 (LI-COR).

### 2.4 Surface and sub-cellular fractionation

To separate cytosolic and membrane fractions of cellular lysates, cells were washed with cold PBS and suspended in buffer containing 320 mM Sucrose, 10 mM TRIS-HCL pH 7.4, 10 mM EDTA and 10 mM EGTA before homogenization using a 26-G needle. Differential centrifugation was then used to isolate subcellular fractions, wherein lysates were spun at 800 × g for 10 minutes to remove nuclei, at 16000 x g for 40 minutes to isolate the cytosolic fraction and then again at 16000 x g for 40 minutes to pellet the membrane fraction. Resulting protein fractions were quantified using the Bradford Assay (B6916 Sigma Aldrich). Following protein quantification, SDS-PAGE separation, and western blotting, GAPDH and Na/K ATPase were used as cytosolic and membrane markers respectively.

To elute cell surface proteins, cells were washed with ice-cold PBS before being incubated, rocking at room temperature with 0.53mM EDTA-PBS for 10 mins, as described in (39) EDTA wash was then collected and subjected to acetone precipitation to concentrate protein amount before being quantified and separated using SDS-PAGE and western blotting. Ponceau staining of total protein was used as a loading control.

### 2.5 Immunoprecipitation

For Annexin A2 immunoprecipitation, cell lysates were pre-cleared with protein G agarose beads (Sigma Aldrich) for 1 hour with gentle rotation at 4°C. Cleared lysates were then transferred in a new tube for incubation with agarose beads and primary anti-Annexin A2 antibody (Rabbit, ab41803, Abcam) overnight at 4°C with gentle rotation. The beads were washed with lysis buffer and boiled with 2x SDS loading buffer at 100°C for 5 minutes before separation using SDS-PAGE.

For fibronectin immunoprecipitation, protein G agarose beads were incubated with anti-FN for 1 hour, rotating at 4°C. Beads were then spun down and added to purified fibronectin-PBS solution for 3 hours, rotating at 4°C. Beads were then washed with lysis buffer and added to MDA-MB-231 cell lysate, and incubated overnight, rotating at 4°C. The beads were washed with lysis buffer and boiled with 2x SDS loading buffer at 100°C for 5 minutes before separation using SDS-PAGE. For all experiments, a control/blank IP was prepared with all the reagents, but without the antibody, to account for non-specific binding.

### 2.6 Mass spectrometry analysis

Following SDS-PAGE, gel lanes containing protein bands stained with Coomassie were excised from the gel and transported to Mass Spectrometry and Proteomics Facility, University of St Andrews, Fife, for analysis as described in (33). Briefly, gel chunks were subjected to trypsin digestion, peptides were separated by nano-LC utilizing an Eksigent two-dimensional LC NanoLC system (Eksigent/Applied Biosystems Sciex, MA, USA) interfaced with a QStar XL mass spectrometer (Applied Biosystems Sciex, MA, USA). Data sets were searched against the NCBInr 20190208 database using MASCOT software (Matrix Science, MA, USA) under the following parameters: maximum one missed cleavage of trypsin digestion, carbamidomethyl (C) as a fixed modification, oxidation (M) as a variable modification, a peptide mass tolerance of ± 20 ppm and a fragment mass tolerance of ±0.05 Da. Only scores higher than the significance threshold (*p* < 0.05) were reported.

### 2.7 Functional and interaction analysis

Prior to functional analysis, protein lists were first reduced using the cut-off of 4 or more peptide matches. Protein-protein interaction networks were probed using STRING v11 (40), visualized using Cytoscape (V3.8.2) with StringApp (41) and analysed using CentiScape app (42). Proteins were then analyzed using the PANTHER Overrepresentation Test (V14.1) (43) with the Fisher’s exact test to calculate p-value and Benjamini–Hochberg procedure to calculate false discovery rate (FDR). FDR cut-off was <0.05. In the case of Cellular Component sub-ontology analysis, REVIGO was used (http://revigo.irb.hr/), at a similarity cut-off of 0.9, to reduce the quantity of redundant and overlapping terms (44). Overrepresented GO terms were then visualized as a network using Cytoscape. Centrality of nodes was assessed using CentiScaPe.

### 2.8 Immunofluorescence of extracellular surface protein

MDA-MB-231 cells were plated at 10,000 cells per well in 96-well Greiner ScreenStar (Cruinn, Dublin, IE) plates and incubated for 24 hours. The cells were then fixed with 4% PFA at room temperature for 10 minutes before PFA was removed and the cells washed with PBS. Fixation was then quenched by the addition of 50mM NH4CL for 1 hour. Cells were not subjected to a permeabilizing agent to maintain membrane integrity and to restrict antibody staining to the extracellular surface. The non-permeabilized cells were then blocked using 2% donkey serum (DS) – PBS. Primary antibodies were then applied, diluted in 10% DS-PBS and incubated overnight at 4°C. The next day, wells were washed three times using PBS, before secondary antibodies and counterstains were added, diluted in 10% DS-PBS. After 1 hour, cells were washed three times with PBS before being covered with PBS for imaging. Plates were then imaged using ImageXpress confocal microscope (Molecular Devices, CA, USA) equipped with metaXpress software. Images were acquired using the 50 μm slit confocal at 20X magnification. 9 sites per well were acquired. Exposure times, focus and offset were maintained for all wells in a plate. Images were then exported and analyzed using Cell Profiler vers.3.1.9 (Broad Institute, US) (45). Within this, to identify primary objects (nuclei) a global threshold strategy, with the Otsu method was used. Secondary objects (cells) were then identified using the Propagation method, under the same threshold parameters.

### 2.9 Use of publically available expression & clinical data

cBioportal (www.cbioportal.org) (46, 47) was used to export expression data and clinical characteristics (as of October 2021) from the Breast Invasive Carcinoma (TCGA, PanCancer Atlas) study. The following search parameters were used: Selected Studies: Breast Invasive Carcinoma (TCGA, PanCancer Atlas) (1084 total samples), Select Genomic Profiles: All, Select Patient/Case Set: All samples (1804). Genes: ANXA2 & COL1A1. Phosphoprotein site level expression data by CPTAC (TMT, Log2ratio) for Tyr 24 of Annexin A2 and COL1A1 mRNA Expression, [RSEM, log2(value + 1)] for 98 samples were plotted against each other using Graphpad Prism and Spearman’s correlation.

## 3 Results

### 3.1 Collagen-I triggers the post-translational phosphorylation of Annexin A2 and subsequent membrane and extracellular translocation in breast cancer cells

**Figure 1:**
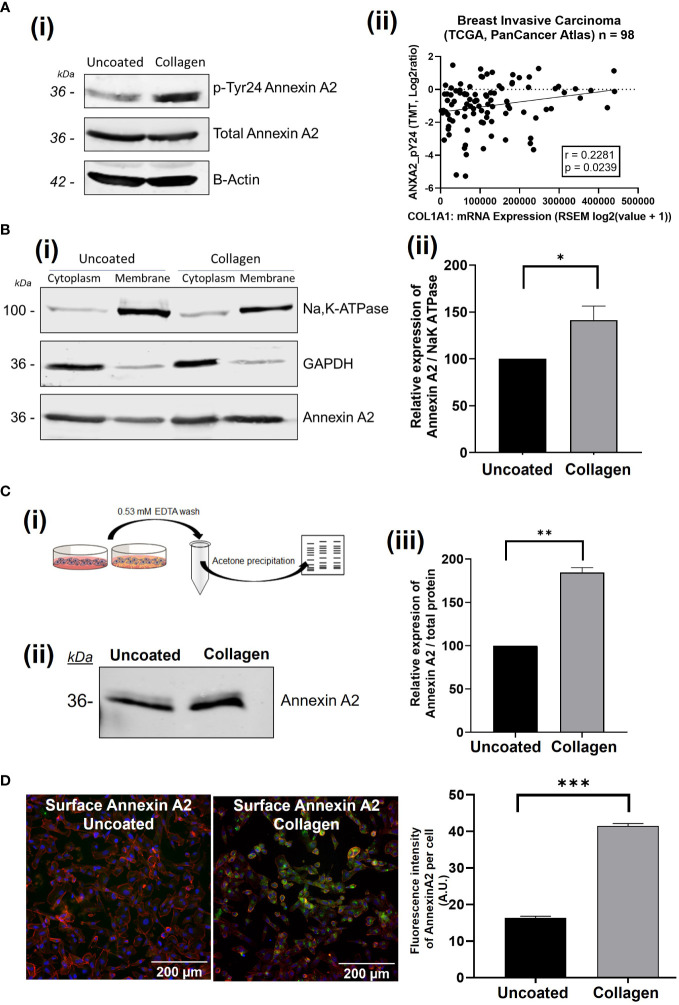
The presence of collagen-I induces the phosphorylation of Annexin A2 at Tyr 24 and translocation to the extracellular membrane. **(A)** MDA-MB-231 cells were grown on uncoated or collagen-I coated plates for 24 hours before western blot analysis. Representative blot and corresponding graph showing the increase in AnxA2 phosphorylation at Tyr24 (n=3). (ii): Scatterplot indicating a significant correlation the relationship between phospho-Tyr24-Annexin A2 levels and COL1A1 expression in breast cancer patient samples from the “Breast Invasive Carcinoma (TCGA, PanCancer Atlas)” study (Spearman’s correlation, r = 0.2429, p = 0.0160, n=98). **(B)** (i) Subcellular fractionation and western blot analysis shows an increase in Annexin A2 at the cell membrane in the presence of collagen-I. GAPDH and Na/K-ATPase were used as markers for the cytoplasm and the membrane respectively. (ii) Densitometry analysis of Annexin A2 protein bands normalized against Na/K-ATPase and expressed as a percentage of Annexin A2 level in uncoated control, measured using Image Studio software. Data displayed as mean +/- SEM. (students T-test, *p < 0.05, n=4) **(C)** (i) Workflow of surface elute quantification. (ii) Western blot showing increased Annexin A2 in the surface elutes of cells plated on collagen-I. (iii) Protein amounts were quantified via densitometry of Annexin A2 protein bands normalized against total protein/ponceau staining and expressed as a percentage of Annexin A2 level in uncoated control. Data displayed as mean +/- SEM. (Student’s T-test, *p < 0.05, n=2). **(D)** Non-permeabilized cells plated on uncoated or collagen-I coated plastic were surface stained for Annexin A2, and counterstained with Hoechst (nuclei) and TRITC phalloidin (actin cytoskeleton), before being imaged and analyzed using Cell Profiler. Analysis of the fluorescence intensity per cell shows a higher fluorescence thus higher surface Annexin A2 expression when cells are plated on collagen for 24 hours. Data displayed as mean integrated fluorescence per cell ± SEM (p = <0.0001, n=3, approx. 3200 cells analyzed per plate). *p ≤ 0.05, **p ≤ 0.01, ***p ≤ 0.001.

Using immunoblot analysis we firstly investigated how the expression and regulation of Annexin A2 in breast cancer cells is affected by the presence of collagen-I. Collagen-I was chosen as it is the most abundant constituent of the ECM and is linked to breast cancer proliferation, migration and metastasis (7, 9, 11, 48). We were interested to see that, in the presence of collagen-I, the phosphorylation of Annexin A2 at Tyr24 is dramatically increased, as illustrated in **Figure 1A**. The protein levels of total Annexin A2 were unchanged. Furthermore, gene expression analysis of ANXA2 mRNA levels shows this increase in phosphorylated Annexin A2 is not a result of increasing total levels of Annexin A2 (**Supplementary Figure 1**), in fact, collagen-I appears to decrease levels of ANXA2 mRNA. This suggests a possible regulation at a translational level, potentially through Annexin A2 phosphorylation promoting the formation of the Annexin A2-S100A10 heterotetrameric complex, in which the proteins have been reported to exert a reciprocal co-stabilization effect on each other’s protein levels (49–51). Moreover, in applying our observation to publically available patient data [Breast Invasive Carcinoma- TCGA, PanCancer Atlas (47)] using cBioportal (46, 52), we can see that there is a low but statistically significant positive correlation between the mRNA expression of COL1A1 and the level of phosphorylation of Annexin A2 at Tyr 24 (Spearman’s correlation, r = 0.2429, p = 0.0160). As seen in **Figure 1A (ii)** this suggests the trend observed in *in vitro* could hold true in patients, pending further investigation from these results, we can conclude that although collagen does not increase the expression of Annexin A2, it does appear to regulate an important post-translational modification of Annexin A2 (23).

Phosphorylation of Annexin A2 at Tyr24 has been shown to modulate AnxA2-S100A10 complex formation and extracellular translocation, as well as cytoskeletal rearrangement and epithelial to mesenchymal transition (18, 21, 53–56). Thus, we investigated how this collagen-I mediated phosphorylation of Annexin A2 affects its location within the cell using a variety of methods. Firstly, using subcellular fractionation to separate whole cell lysates into cytosolic and membrane fractions, we showed that when cells are plated on collagen-I, more Annexin A2 is found within the membrane fraction than cells on uncoated plastic. (**Figure 1B**, Student’s T-test, p = 0.0356, n=3).

As Annexin A2 relies on Ca^2+^ for its membrane phospholipid binding properties, the addition of a chelating agent such as EDTA can disrupt this binding and elute Annexin A2 from the surface of cells. We next wanted to test whether the presence of collagen-I would affect the amount of Annexin A2 eluted. To do this, cells were plated on either uncoated or collagen-I coated 10cm^2^ plates, incubated for 24 hours then washed with EDTA-PBS. As this wash was extremely dilute, we carried out an acetone precipitation to concentrate the protein amount before running on 12% SDS-PAGE and western blotting. As shown in **Figure 1C**, a greater amount of Annexin A2 protein was eluted from the cell surface when the cells were plated on collagen-I (Student’s T-test, p = 0.0042, n=2). The amount of Annexin A2 was normalized to total protein content using ponceau staining and Image studio software.

To further confirm this movement of Annexin A2 to the extracellular surface of cells when in contact with collagen-I we next employed immunofluorescent microscopy. Cells were plated on either uncoated or collagen-I coated wells for 24 hours before being fixed and surface stained for the presence of Annexin A2. The omission of a permeabilization agent, which penetrates the cell membrane, allowed us to label the extracellular Annexin A2. Following acquisition, images were imported into Cell Profiler for single cell fluorescence analysis, with the workflow outlined in **Supplementary Figure 2**. Comparison of the fluorescence intensity of Annexin A2-A488 surface staining showed that non-permeabilized cells plated on collagen expressed more surface Annexin A2 than non-permeabilized cells plated on plastic. Statistical analysis shows the mean fluorescence of cells on collagen-I is significantly higher than cell on plastic (**Figure 1D**), Student’s t test, p = <0.0001, n=3). These results strongly suggest, through three independent experimental methods, that the presence of collagen-I triggers the cell surface localization of Annexin A2 in breast cancer cells.

### 3.2 The identification of candidate Annexin A2 binding proteins using interactome analysis

**Figure 2:**
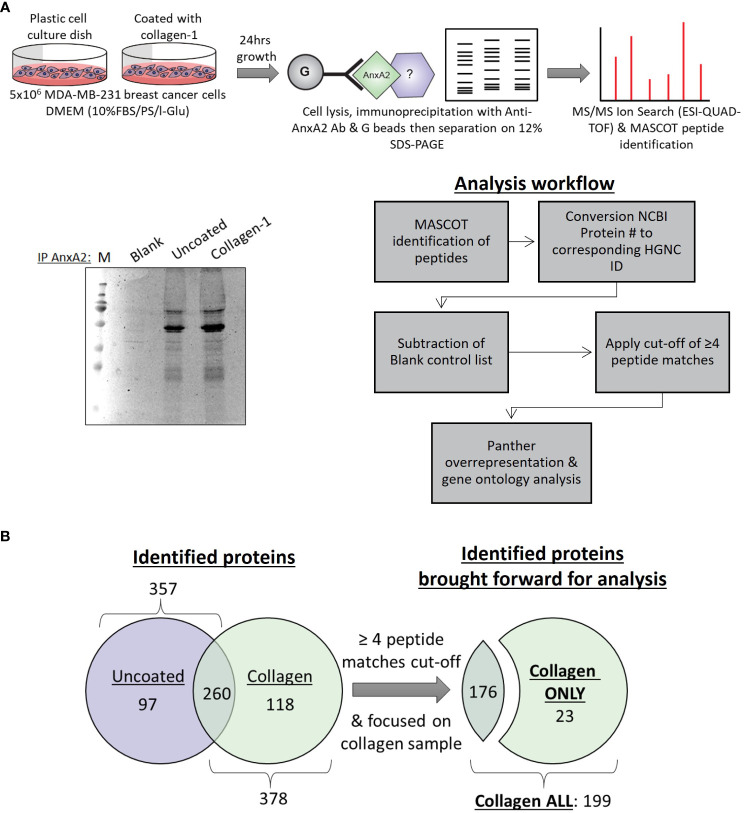
Mass spectrometry analysis of Annexin A2 binding partners with and without the presence of collagen-I **(A)** Optimization and setup of a workflow to determine Annexin A2 binding partners and how this is affected by the presence of collagen-I. **(B)** Lists of identified proteins from “Uncoated” (n=357) and “Collagen” (n=378) samples were truncated according to the cut-off of ≥ 4 peptide matches, producing “Collagen ONLY” (n=23) and “Collagen ALL” (n=199) lists for subsequent analysis.

Following our discovery of the role collagen-I plays in the post-translational phosphorylation and localization of Annexin A2, our next step was to investigate the functional consequences of this relationship in cancer cells. To achieve this, we investigated Annexin A2 interactors and how this is altered when cells are in contact with collagen-I. Protein-protein interaction analysis is a well-known method of investigating protein function and signal transduction within cells (57). To investigate the interactions of Annexin A2 in our cells, we carried out an Annexin A2 affinity pull-down, followed by a mass spectrometry screen as illustrated in **Figure 2A**. The proteins identified in the “Blank” or no antibody, negative control sample were subtracted from the “Uncoated” and “Collagen” binding partners lists to account for unspecific binding and contaminates. This resulted in lists of 357 proteins in the “Uncoated” sample and 378 proteins in the “Collagen” sample being identified as candidate Annexin A2 binding proteins. Each protein was assigned its corresponding HGNC gene id to avoid any duplicates, protein aliases or Microsoft Excel errors being included (58).

In both lists, Annexin A2 was one of the most highly scored proteins, giving us confidence in the reliability and quality of our experimental approach and adding credence to our subsequent findings. Furthermore, previously reported binding partners of Annexin A2 such as Ahnak (59, 60) and RACK1(aka GNB2L1) (61, 62) were also identified and highly scored within our lists further adding to experimental validity. To ensure the validity of identified proteins and to maintain a high level on confidence in our findings, only proteins with ≥ 4 peptide matches were included in subsequent analysis from here on, as displayed in **Figure 2B**.

**Figure 3:**
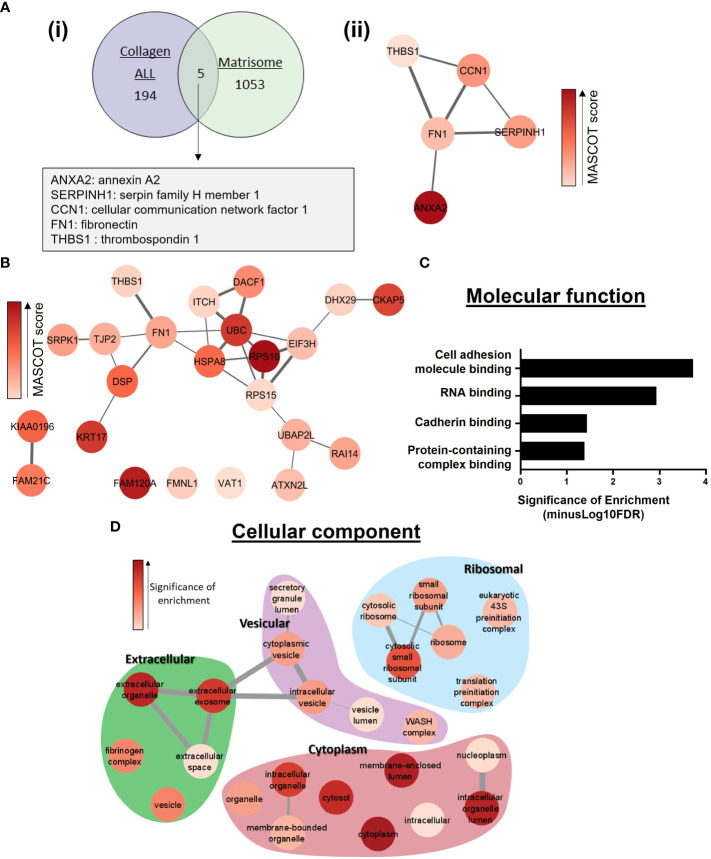
Functional enrichment analysis of proteins identified as Annexin A2 binding partners in the presence of collagen-I. **(A)** (i) Comparison of the overlap between identified proteins within “collagen-I ALL” list and constituents of the extracellular matrix, derived from the MatrisomeDB (63) (ii) Protein-protein interaction network of the 5 proteins identified in (i). Interactions were assessed with String (40) and visualized using the StringApp in Cytoscape set at 0.4 confidence level (41). Node coloured indicates MASCOT (MS/MS) score. Edge line thickness denotes the confidence of association between protein nodes**. (B)** Protein-protein interaction network of the 23 proteins identified in the “collagen-I only” list. Assessed as in **(A)** (ii), with 0.3 confidence level**. (C)** Functional enrichment analysis of Annexin A2 binding partners in “collagen-I ONLY” was carried out using the PANTHER overrepresentation test (43). Molecular Function overrepresentation test shows cell adhesion molecule binding to be the most significantly enriched term in the list of identified proteins. **(D)** Cellular Compartments overrepresentation test shows a wide range of significantly overrepresented GO terms, including several extracellular associated terms.

Due to our discovery of the role of collagen-I in regulating the post-translational modification and location of Annexin A2, we assessed the overlap between proteins identified as Annexin A2 interactors when cells are plated on collagen-I (referred to as “Collagen ALL”) and proteins known to be involved in the ECM. This was achieved by comparing with the MatrisomeDB human *in-silico* matrisome dataset, which is a curated collection of proteomic data from 17 studies on the ECM (63). The comparison (**Figure 3A**), revealed a discrete and interconnected network of proteins, centered around the ECM protein fibronectin (FN1). Analysis of this network using Centiscape (42) showed FN1 to have the maximum values of centrality parameters including: eccentricity, closeness, centroid, betweenness, stress and radiality, suggesting a central regulatory role of FN1 within this network (**Figure S3**). To further probe the specific effect of collagen-I on Annexin A2, we next evaluated the proteins which were only identified in the collagen-I sample (i.e., subtracting those found in the intersection with “Uncoated” control), hereafter named “Collagen ONLY”, (**Supplementary Table 2**). When we investigate the protein-protein interactions within this list using String, we can see that nearly every node is in an interconnected network, again surrounding fibronectin as a central node, even without the addition of Annexin A2 as a central bait node (**Figure 3B**). This suggests a functional relationship or pathway between these potential Annexin A2 binding partners, with fibronectin as a central interactor. Furthermore, analysis of fibronectin’s position of centrality within the network revealed high values for a number of parameters, including radiality (protein is easily central to the regulation of other proteins but with the possibility to be irrelevant for few other proteins), stress (protein is highly relevant in connecting regulatory molecules and heavily involved in cellular processes) and betweenness (likely crucial to maintain functionality and coherence of signaling mechanisms) (**Supplementary Figure 3A**) (42, 64). In addition, a scatter plot of node centrality highlights the nodes of most relevance in the upper right quadrant as FN1, followed by UBC and RPS15 (**Supplementary Figure 3B**). To understand the functional consequences of collagen-I’s effect on the regulation of Annexin A2, we further analyzed the “Collagen ONLY” list of proteins, using the PANTHER overrepresentation test (43). As depicted in **Figure 3C** the most significantly enriched function was cell adhesion molecule binding (GO:0050839), with 8 of the identified proteins being associated with this GO term (Desmoplakin, Thrombospondin-1, DExH-Box Helicase 29, Heat Shock Protein Family A (Hsp70) Member 8, Fibronectin, Tight junction protein ZO-2, Ataxin-2-like protein and Cytoskeleton-associated protein 5). The Gene Ontology term “cell adhesion molecule binding” is defined as interacting selectively and non-covalently with a cell adhesion molecule or adhesive extracellular matrix constituent (65). This again points to fibronectin as an important interactor of Annexin A2 within the context of ECM regulation. Furthermore, several extracellular associated cellular compartments were found to be significantly overrepresented within the “Collagen ONLY” list (**Figure 3D**). This was of great interest to our study due to our focus on the relationship between Annexin A2 and the extracellular environment, particularly the ECM. This result suggests that when cells are in contact with collagen-I, Annexin A2 is more likely to be involved in ECM binding, in particular fibronectin. This is consistent with our observations that in this state, Annexin A2 is phosphorylated at Tyr 24 and is also translocated to the extracellular membrane.

### 3.3 Reduction of Annexin A2 expression decreases the ability of breast cancer cells to degrade fibronectin and this ability is influenced by the presence of collagen-I

**Figure 4:**
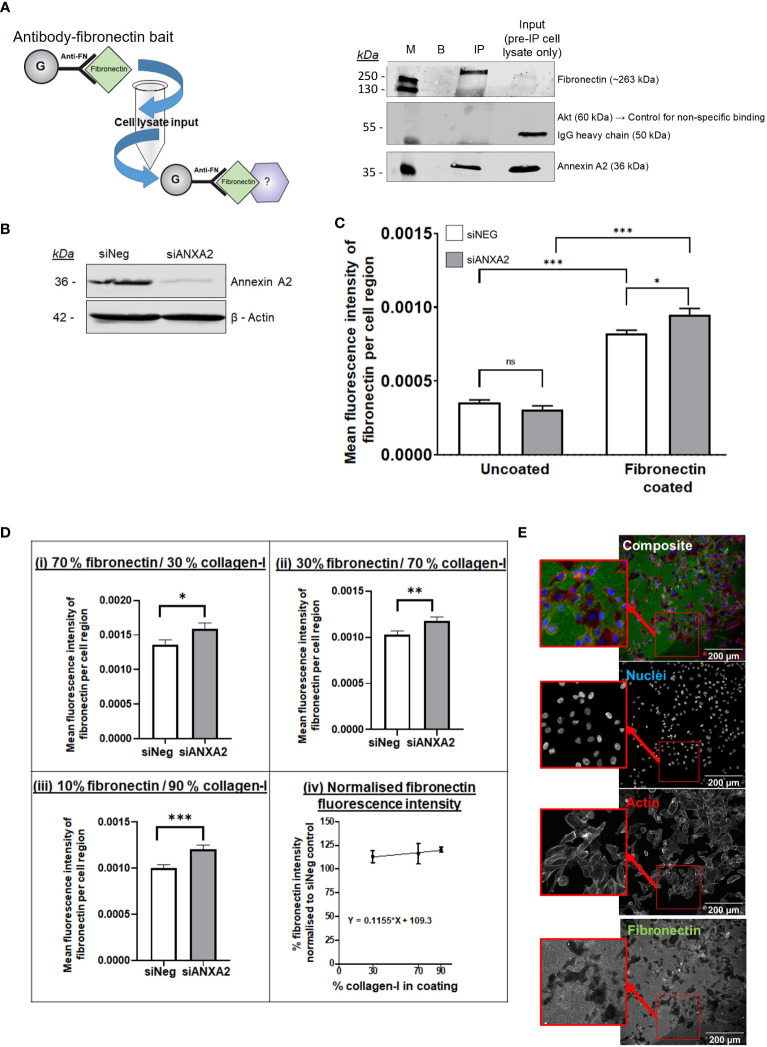
Annexin A2 is capable of binding to fibronectin and reduction in Annexin A2 expression reduces the ability of cells to degrade fibronectin, an ability that is further influenced by the presence of collagen-I. **(A)** Immunoprecipitation of Annexin A2 binding to Fibronectin. Pull-down bait of exogenous purified fibronectin was prepared and incubated with MDA-MB-231 cell lysate. Western blot demonstrating the binding between fibronectin and Annexin A2 using fibronectin immunoprecipitation in MDA-MB-231 lysate. M= Protein size marker, B= Blank/No Ab control, IP= Anti-FN coupled with purified fibronectin then cell lysate, input= MDA-MB-231 cell lysate used for IP before incubation with FN bait. **(B)** Representative blot showing efficacy of Annexin A2 siRNA knockdown. **(C)** 72 hours after transfection, cells were plated in wells coated with fibronectin and then incubated for 24 hours before being stained for Fibronectin. Fibronectin coating correlated with a significant increase in mean fluorescence intensity per cell. For cells on fibronectin coated plastic, KD of Annexin A2 resulted in increased fibronectin staining intensity, showing a decreased ability of cells to degrade fibronectin. (Students T test, *p < 0.05, ***p <0.0001, n =3) **(D)** For cells on defined mixtures of fibronectin and collagen-I coatings, KD of Annexin A2 resulted in increased fibronectin staining intensity, the difference between KD and siNeg control increased as the proportion of collagen-I was increased. (Mann-Whitney U, *p < 0.05, **p < 0.001, ***p < 0.0001, n =3) **(E)** Representative immunofluorescent images of fibronectin degradation quantification. ns, not significant.

Given the results of our mass spectrometry analysis identifying fibronectin as a novel Annexin A2 binding partner of central importance (**Figure 3B**), we decided to further investigate the relationship between the two proteins. We first tested their binding directly by carrying out a fibronectin baited immunoprecipitation of Annexin A2, verifying the binding between the two proteins (**Figure 4A**; **Supplementary Figure 4**). Having confirmed the interaction between Annexin A2 and fibronectin, we next examined its functional consequence. It is reported that when Annexin A2 becomes phosphorylated and translocated to the extracellular surface, it acts as an activation platform for plasmin, a protease known to directly degrade ECM proteins including fibronectin (27, 66, 67). We hypothesized that Annexin A2 could regulate the degradation of fibronectin, as a known interactor of both plasminogen and its activators (27, 29), and a novel interactor of fibronectin. To test our hypothesis, we assessed the direct effect of Annexin A2 expression on the degradation of fibronectin using a modified gelatin degradation assay (68) and high-throughput immunofluorescent imaging. Cells were transfected with siANXA2 or Negative control siRNA and grown for 72 hours to allow the knockdown to take effect (**Figure 4B**). Cells were then plated on uncoated wells, fibronectin coated wells or on wells coated with various percentages of fibronectin and collagen-I combinations. Degradation of fibronectin was detected as a localized loss of fluorescence (as illustrated in **Figure 4E**; **Supplementary Figure 5**). To quantify this, Cell Profiler was used to identify cells within each image, and the mean fluorescence intensity of fibronectin staining per cell was then measured.

From **Figure 4C**, we can see that coating the wells with fibronectin gives a large increase in the measured fluorescence compared to uncoated wells, giving us confidence that our approach was appropriate to quantify fibronectin abundance. Cells with reduced Annexin A2 expression had significantly lower ability to locally degrade fibronectin, as demonstrated by a marked increase in mean fluorescence within each cell area (Student’s T test, p = 0.0110). From this we can conclude that a reduction in Annexin A2 reduces the cells ability to degrade fibronectin, likely via Annexin A2’s known role in plasmin activation.

We next examined the effect of plating cells on defined mixtures of collagen-I (COL) and fibronectin (FN) to assess whether the presence of collagen-I could influence the Annexin A2 mediated digestion of fibronectin (**Figure 4D**). Using majority fibronectin coating of 70% FN/30%COL, we can see that at these proportions, the reduction of Annexin A2 expression causes a significant increase in measured fibronectin abundance (Mann-Whitney U test, p = 0.0276). This effect continues as the loss of Annexin A2 consistently reduces the average fluorescence of stained fibronectin for 30% and 10% fibronectin coating (**Figure 4D (iv)**). Remarkably, the significance of this difference actually increases when the coating mixture is predominantly collagen-I, suggesting a more dramatic effect with more collagen-I present. This can be seen in **Figure 4D** for 30%FN/70%COL (Mann- Whitney U, p = 0.0052) and for 10%FN/70%COL (Mann-Whitney U, p = 0.0006). To compare the result of ANXA2 knockdown across the different coating ratios, the fluorescence intensity of fibronectin in the ANXA2 knockdown condition was normalized relative to the fluorescence intensity of fibronectin in the negative control siRNA condition for each plate. As illustrated in **Figure 4D (v)**, the relative % increase in fibronectin fluorescence intensity does increase slightly as the ratio of collagen-I within the coating increases, as measured by a positive value for the slope of the line. This suggests that Annexin A2 expression has a dominant effect on the cell’s ability to degrade fibronectin. When Annexin A2 is knocked down, the cells’ ability to degrade fibronectin does not increase with increase in collagen-I abundance. These results suggest the effect on the cells ability to degrade fibronectin is dependent on high expression of Annexin A2 rather than merely collagen-I abundance.

## 4 Discussion

In this study, we have shown that the post-translational modification, localization and functional interactions of Annexin A2 are strongly influenced by the ECM, in particular, collagen-I, the most abundant collagen type in the body (69, 70). We firstly observed that collagen-I stimulates the phosphorylation of Annexin A2 at Tyr 24 and, accordingly, also triggers the translocation of Annexin A2 to the extracellular membrane. High collagen-I conditions are pertinent in the study of breast cancer as collagen-I is often overexpressed and associated with poor clinical outcomes (2, 6, 36, 71). This post-translational modification at Tyr24 is of particular interest in malignancy due to the early discovery of phosphorylated Annexin A2 in transformed cells (72, 73) and more recent observations of increased Tyr24 phosphorylation levels in cancer (74). Furthermore, a number of studies have linked phosphorylation of Annexin A2 to the promotion of EMT and metastatic cell behaviors in a variety cancer types (21, 53, 54, 61, 75–78). Our observed link between Tyr24 and collagen-I is particularly interesting given the independent observations that both Tyr24 phosphorylation (21) and collagen-I (7) can increase cell migration. This suggests Annexin A2 may be integral in the pathways contributing to collagen-I associated malignancy and cell migration. Moreover, the use of a specific imaging marker targeted to phospho-ANXA2 on a variety of solid tumor types has shown Annexin A2 is highly phosphorylated in the TME, with particular localization at the invasive tumor front (56). In addition, this study showed both cancer cells and cancer associated fibroblasts express phosphorylated Annexin A2, whereas normal fibroblasts do not. Given the integral role of cancer associated fibroblasts in the production of a desmoplastic TME, and the aberrant remodeling of ECM proteins such as collagen-I and fibronectin, our observations that collagen triggers the phosphorylation of Annexin A2 provides potential explanation for the specific upregulation of phospho-Annexin A2 at Tyr 24 on tumor cells and within the TME seen in the study by Shen et al. (56). Furthermore, our evidence of a link between collagen abundance and the activation of this pro-metastatic modification of Annexin A2 goes some way to explaining our previous findings of Annexin A2’s integral role in breast cancer metastasis and the associated ECM dysregulation seen in the disease (33).

We next conducted a protein interactome analysis which yielded an extensive list of potential binding partners of Annexin A2. Knowing collagen-I triggers the phosphorylation and translocation of Annexin A2, we hypothesized that investigating Annexin A2 interactors under these conditions may provide insight into the functional consequences of this regulation. Of note within the list of proteins found bound to Annexin A2 only when cells are plated on collagen-I (**Table S1**) is Keratin 17, which was previously discovered to be a novel binding partner of Annexin A2 in a study by Chung et al. (79). This study found that when Keratin-17 expression is silenced, the phosphorylation of Annexin A2 at Tyr 24 is reduced, with the authors hypothesizing that keratin filaments act as a scaffold to regulate the subcellular location of Annexin and facilitate its phosphorylation. This may provide an explanation for our observation that Keratin-17 is bound to Annexin A2 only when our cells are plated on collagen-I as this is when we see an increase in Tyr24 phosphorylation. Once again, this agreement of our findings with the published literature provides strong validity for our approach and hypothesis that the collagen-I mediated regulation of Annexin A2 affects its interactome, with potential functional consequences.

Thus, in attempting to elucidate the effect of collagen-I on Annexin A2 function, we assessed the specific proteins found bound to Annexin A2 in this condition, particularly in terms of the ECM. This revealed a network of interacting proteins with fibronectin, an ECM constituent and glycoprotein, emerging as a central and important node. Furthermore, gene ontology enrichment analysis revealed cell adhesion molecule binding, also known as adhesive extracellular matrix constituents, to be the most significantly overrepresented function with our identified list, an ontology of which fibronectin is a member. Although not expressed in healthy adult breast tissue, fibronectin expression is increased in breast tumor stroma (80) and has been linked to the promotion of proliferation, angiogenesis and metastasis of breast cancer cells (81, 82). To our knowledge, the interaction between Annexin A2 and fibronectin has not been characterized. Using String (40) to search for published evidence of PPIs, one study was found that suggested Annexin A2 as a potential interactor due to co-precipitation with associated α5β1 integrin complexes (83). Furthermore, inhibition of Annexin A2 interactions using a specific targeted peptide reduces the adhesion of cells to fibronectin (84). Given the role of Annexin A2 in promoting breast cancer progression *in vivo* and in patients evidenced by us (33) and several others, coupled with our observation that proteins involved in cell adhesion to the ECM, including fibronectin, are enriched in our list of Annexin A2 binding partners, it is reasonable to hypothesize that the collagen–I regulation of Annexin A2 may influence its role in degradation and remodeling of the ECM.

A critical hallmark of malignancy is its capacity to spread and invade neighboring tissue. This metastatic ability is facilitated by the proteolytic cleavage of ECM proteins that make up physiological barriers like the basement membrane, upon intra- and extravasation (85–87). It is well evidenced that cell-surface Annexin A2 promotes the localized activation of plasmin to regulate vascular fibrinolysis and neoangiogenesis (32, 88–92). Plasmin, a serine protease, has been shown to degrade fibronectin both *in vitro* (93, 94) and *in vivo* (95, 96). Within the context of cancer, plasmin can proteolytically remodel the cancer associated ECM (including fibronectin), creating a path which enhances cancer cell escape from the primary site (26, 97, 98). Thus, to characterize the implications of Annexin A2 binding to fibronectin, we examined whether Annexin A2 expression specifically affects the degradation of fibronectin by breast cancer cells, something that has not been directly investigated before. Remarkably, we saw that knockdown of Annexin A2 did in fact impair the fibronectin degradation ability of MDA-MB-231 breast cancer cells. This is of critical relevance in breast cancer progression as fibronectin turnover has been shown to increase the metastatic capacity of tumor cells (99–101). Furthermore, the proteolytic cleavage of fibronectin by plasmin creates fibronectin degradation products that further enhance the migration and invasion of tumor cells, through their interaction with cell surface integrin receptors (97, 98). This suggests Annexin A2 has an integral role in the degradation of fibronectin by cancer cells and modulating Annexin A2 activity could be a viable therapeutic strategy, in the yet unsuccessful targeting of plasmin-mediated cancer cell dissemination (102, 103). Further investigation of this mechanism using known pharmacological inhibitors of Annexin A2 phosphorylation, such as Src targeting agents (61) or a combination of phospho-null or phospho-memetic expressing cells in 3-dimensional culture or *in vivo* models may provide insight into future clinical targeting potential.

Taken together, our work shows that the regulatory effect of collagen-I on the phosphorylation and subsequent localization of Annexin A2, and in enhancing Annexin A2’s pro-tumorigenic role in the degradation of fibronectin. Bearing in mind the association of collagen-I over-expression with migration, invasion, breast cancer metastasis and poor survival (36), we can hypothesize that collagen-I’s influence on Annexin A2 phosphorylation and the subsequent degradation of fibronectin, may be one of the many ways in which the ECM can promote cancer progression. Furthermore, these findings go some way to explain the molecular mechanisms and associated biological processes that Annexin A2 phosphorylation mediates in the TME, and how these are plausibly connected to aggressive disease and cancer metastasis, as seen in our previous work, and that of others (21, 31, 33, 35, 61).

## 5 Conclusions

Ultimately, we have shown that the phosphorylation, location and functionality of Annexin A2 in cancer is regulated by the TME. In addition, we have greatly increased our knowledge of the function of Annexin A2 in breast cancer cells, through interactome studies, contributing to the overall understanding of this protein. Our work demonstrates that Annexin A2 has a distinct role in mediating the dynamic reciprocity between the ECM and cancer cells. The expression, localization and interactions of Annexin A2 allow it to function as a bi-directional negotiator between the extracellular environment, the cancer cell and back out to the TME. Our findings suggest a mechanism for the poor clinical outcomes associated with a desmoplastic TME as we hypothesize that the high expression of collagen-I seen in desmoplasia can increase the invasive capacity of breast cancer cells via its regulation of Annexin A2. Further investigations with additional cancer cell lines, primary cancer cells and *in vivo* models are necessary to identify specific clinical disease manifestations in which Annexin A2 and collagen-I interactions influence the cancer phenotype and could be targeted in the prevention of cancer progression. Our previous findings showing the association of Annexin A2 expression with metastatic progression, combined with our new observations of how Annexin A2’s role is heavily influenced by the ECM points to the interaction between Annexin A2 and collagen-I as a target of interest in cancer progression. Future studies to unpick the precise molecular mechanism and highlight targetable nodes within this pathway are needed to translate these findings to breast cancer patients. Given the major role of ECM degradation in cell migration and invasion, this is an obvious avenue of investigation for anti-cancer therapies. However, due to the poor results to date from clinical trials of proteolysis inhibitors such as Batimastat, Amiloride and Upamostat (102–104), it is clear a new and perhaps indirect, strategy is needed in preventing cancer metastasis. Thus, we suggest the targeting of Annexin A2 to prevent its translocation or protein interactions may be an approach to halting the ECM and TME mediated progression of breast cancer.

## Data availability statement

The proteomics data presented in the study are deposited in the MassIVE ProteoSAFe repository, accession number MSV000093084, available at: https://doi.org/doi:10.25345/C50R9MF2R. Further inquiries can be directed to the corresponding authors.

## Ethics statement

Ethical approval was not required for the studies on humans as only commercially available established cell lines were used and publicly available data from anonymised online databases was used. No animal subjects were used in this study.

## Author contributions

AM: Conceptualization, Formal analysis, Investigation, Methodology, Visualization, Writing – original draft. JN: Formal analysis, Visualization, Writing – review & editing. ROC: Formal analysis, Visualization, Writing – review & editing. AL: Conceptualization, Supervision, Writing – review & editing. JA: Project administration, Writing – review & editing. PK: Conceptualization, Funding acquisition, Supervision, Writing – original draft. KM: Conceptualization, Formal analysis, Funding acquisition, Investigation, Methodology, Supervision, Visualization, Writing – original draft.
