# Supplementary material for: Collagen-I influences the post-translational regulation, binding partners and role of Annexin A2 in breast cancer progression
Source: Front Oncol. 2023 Oct 24;13:1270436. doi: 10.3389/fonc.2023.1270436 (PMC10628465; doi:10.3389/fonc.2023.1270436)
Supplement: Supplementary file 1 [file DataSheet_1.pdf]

Supplementary Material

Collagen-I influences the post-translational regulation, binding partners and role of Annexin A2 in breast cancer progression

1 Supplementary Tables

Supplementary Table 1: List of antibodies used in western blotting, immunofluorescence and immunoprecipitation in paper.

Target	Cat number	Species	Source	Usage	Ref in text
Phospho-Annexin A2 (Tyr24)	sc-135753	Mouse	Santa Cruz Biotechnology Inc.	Western blot - 1:250.	Figs: 1(A)
Annexin A2	ab41803	Rabbit	Abcam	Western blot – 1:1000.<br>Immunofluorescence – 1:500<br>Immunoprecipitation - 1 µg per 500 µg of total protein	Figs: 1(A, C, D), 4 (A, B)
Annexin A2	610069	Mouse	BD Transduction Laboratories	Western blot – 1:1000	Figs: 1(B)
Beta-Actin	A5441	Mouse	Sigma Aldrich Ltd	Western blot – 1:1000	Figs: 1(A), 4(B)
GAPDH	SAB1405848	Mouse	Sigma Aldrich Ltd	Western blot – 1:1000	Figs: 1(B)

Na/K-ATPase	30105S	Rabbit	Cell Signalling Technologies	Western blot – 1:1000	Figs: 1(B)
Fibronectin	sc-8422	Mouse	Santa Cruz Biotechnology Inc.	Immunoprecipitation - 2 µg per 100 µg of total<br>Protein.<br>Western Blot- 1:100<br>Immunofluorescnce -1:100	Figs: 4 (A, C, D, E)
Akt	9272S	Rabbit	Cell Signalling Technologies	Western Blot – 1:1000	Fig 4 (A)

Supplementary Table 2: List of candidate Annexin A2 binding partners identified only when MDA-MB-231 cells are plated on a collagen-I substrate.

Protein name	Gene	HGNC	UniProt	Brief Description
Keratin 17	KRT17	6427	Q04695	Type I cytokeratin. Upreg. Via EGFR activation and reported binding partner of Annexin A2.[1]
Ribosomal protein S16	RPS16	10396	P62249	Component of the small 40s subunit of the mammalian ribosome.

Cytoskeleton Associated Protein 5	CKAP5	28959	Q14008	Regulates microtubule dynamics organization and plays a role in spindle formation.
Ubiquitin C	UBC	12468	P0CG48	A polyubiquitin precursor.
Family With Sequence Similarity 120A<br><br>or<br><br>Oxidative Stress-Associated Src Activator	FAM120A	13247	Q9NZB2	Involved in oxidative stress-induced survival signaling. Activates src family kinases and acts as a scaffolding protein for the phosphorylation of PI3-K.
Desmoplakin	DSP	3052	P15924	Involved in the organization of the desmosomal cadherin-plakoglobin complexes into discrete plasma membrane domains and in the anchoring of intermediate filaments to desmosomal plaques.
WASH Complex Subunit 5	KIAA0196	28984	Q12768	Recruits complexes to induce actin polymerization at endosomal surfaces, and

				plays a key role in the fission of tubules.
DDB1 And CUL4 Associated Factor 1	DCAF1	30911	Q9Y4B6	Involved in ubiquitin-protein ligase complexes and as an atypical serine/threonine-protein kinase. Known to play key roles in processes such as cell cycle, telomerase regulation and histone modification.
WASH Complex Subunit 2C	FAM21C	23414	Q9Y4E1	Similar to WASHC5, mediates the recruitment of the F-actin-capping protein dimer to the WASH core complex.
Heat Shock Protein Family A (Hsp70) Member 8	HSPA8	5241	P11142	Constitutively expressed heat-shock protein, functions as a chaperone, and binds to nascent polypeptides to facilitate correct folding.
Retinoic Acid Induced 14	RAI14	14873	Q9P0K7	Associated with cortical actin cytoskeleton structures in cell-cell adhesion sites and stress fibres.

Thrombospondin-1	THBS1	11785	P07996	Adhesive glycoprotein that mediates cell-to-cell and cell-to-matrix interaction.
SRSF Protein Kinase 1	SRPK1	11305	Q96SB4	Involved in the regulation of mRNA splicing by regulating localization of splicing factors.
DExH-Box Helicase 29	DHX29	15815	Q7Z478	A member of the DEAD box family of RNA helicases, that functions in translation initiation.
Vesicle Amine Transport 1	VAT1	16919	Q99536	An integral membrane protein of cholinergic synaptic vesicles, thought to be involved in vesicular transport.
Ubiquitin Associated Protein 2 Like	UBAP2L	29877	Q14157	Involved in the ubiquitin–proteasome system and aggregate formation induced by proteasome inhibitors.[2]
Fibronectin	FN1	3778	P02751	Extracellular matrix protein involved in cell adhesion and migration.

Ribosomal Protein S15	RPS15	10388	P62841	Component of the small 40s subunit of the mammalian ribosome.
Tight junction protein ZO-2	TJP2	11828	Q9UDY2	A component of the tight junction barrier in epithelial and endothelial cells.
Formin Like 1	FMNL1	1212	O95466	Required in the cortical actin filament dynamics and cell shape.
Itchy E3 Ubiquitin Protein Ligase	ITCH	13890	Q96J02	Acts as an E3 ubiquitin-protein ligase, involved in targeting proteins for lysosomal degradation.
Ataxin 2 Like Protein	ATXN2L	31326	Q8WWM7	Involved in the regulation of stress granule and P-body formation.
Eukaryotic Translation Initiation Factor 3 Subunit H	EIF3H	3273	O15372	Part of the eukaryotic translation initiation factor 3 (eIF-3) complex, required for in translation initiation.

Note: Description information adapted from Stelzer *et al.* (2016).

Additional References : [1] : Chung *et al.* (2012) [2]: Huang *et al.* (2020)

2 Supplementary Figures

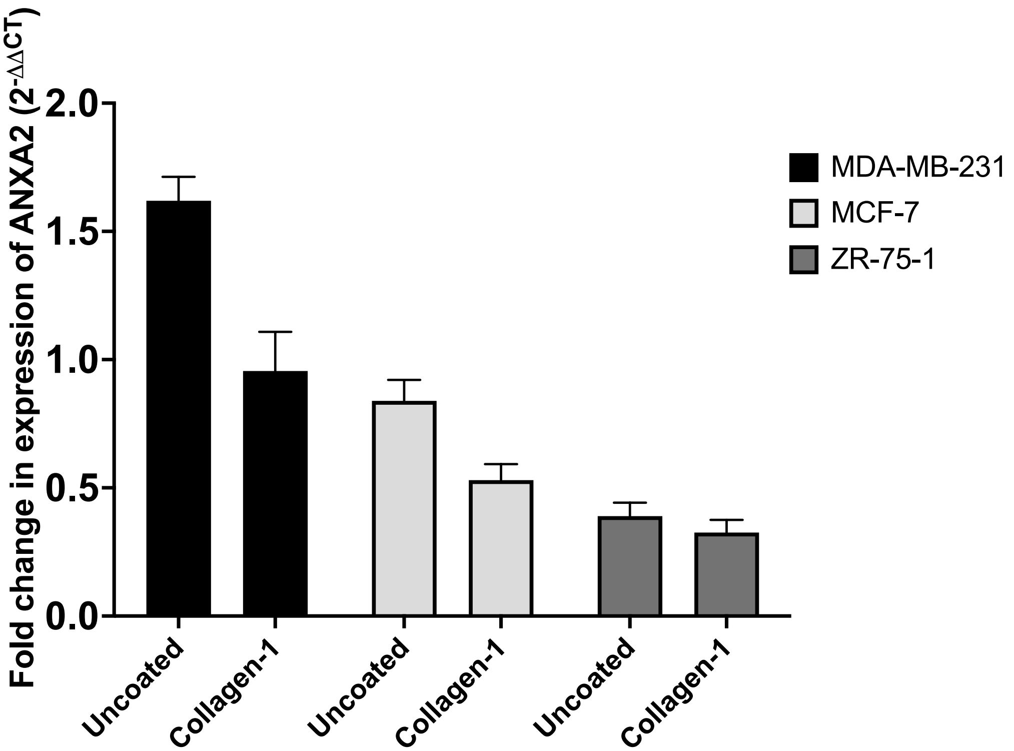

Supplementary Figure 1: qPCR analysis of ANXA2 expression in breast cancer cell lines when cultured on collagen-I coated plates. Gene expression of ANXA2 in three breast cancer cell lines: MDA-MB-231, MCF-7 and ZR-75-1. Expression was calculated using delta CT method and displayed as fold change (corrected RQ) in comparison to uncoated MCF-7 expression (i.e. mean uncoated MCF-7 expression normalised to 1) ±SEM. Differences between cell lines analysed by students T test. **(***= $p<0.05$, n=3)

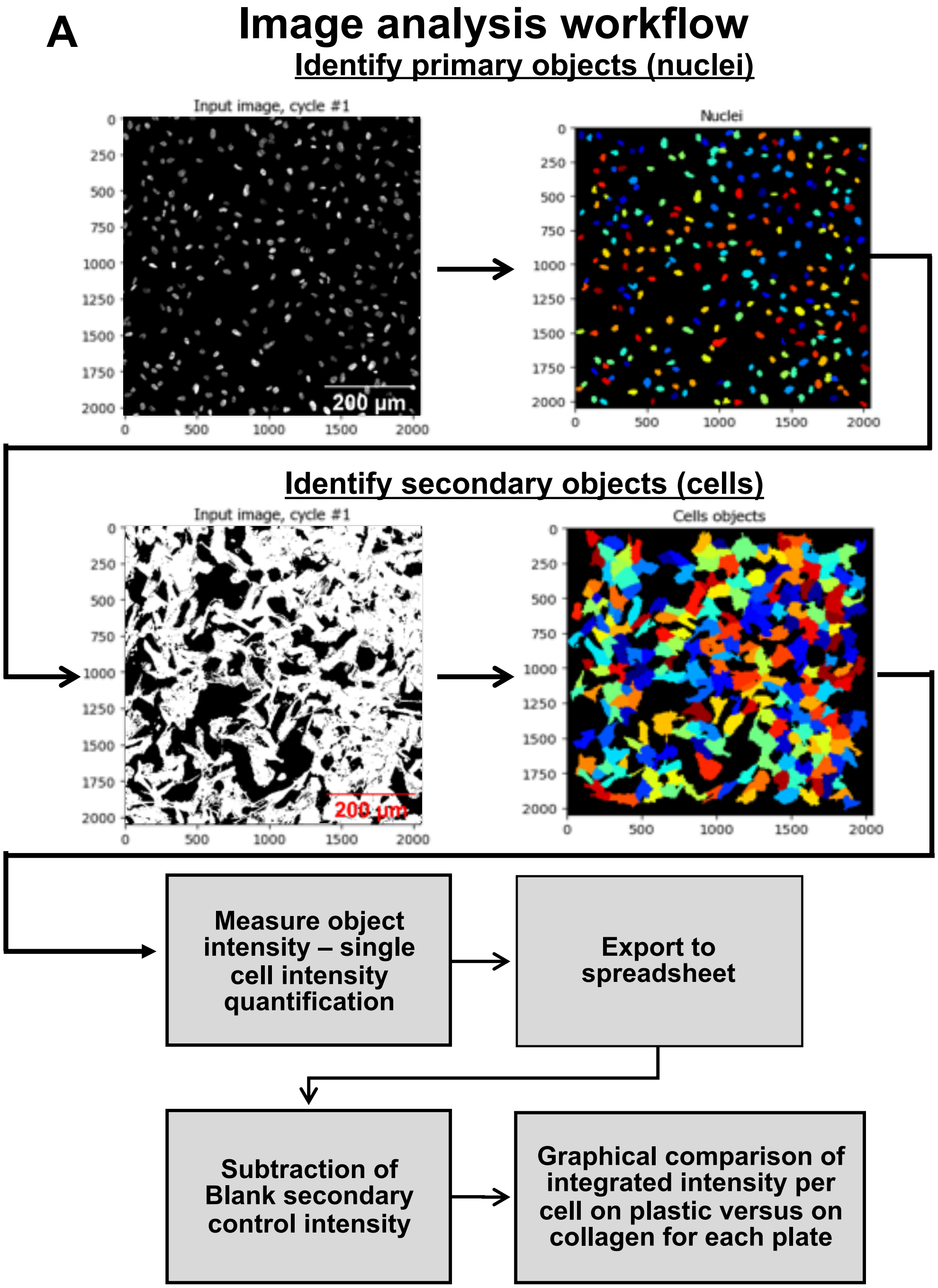
A
Image analysis workflow
Identify primary objects (nuclei)
Input image, cycle #1
200 µm
Nuclei
Identify secondary objects (cells)
Input image, cycle #1
200 µm
Cells objects
Measure object intensity – single cell intensity quantification
Export to spreadsheet
Subtraction of Blank secondary control intensity
Graphical comparison of integrated intensity per cell on plastic versus on collagen for each plate

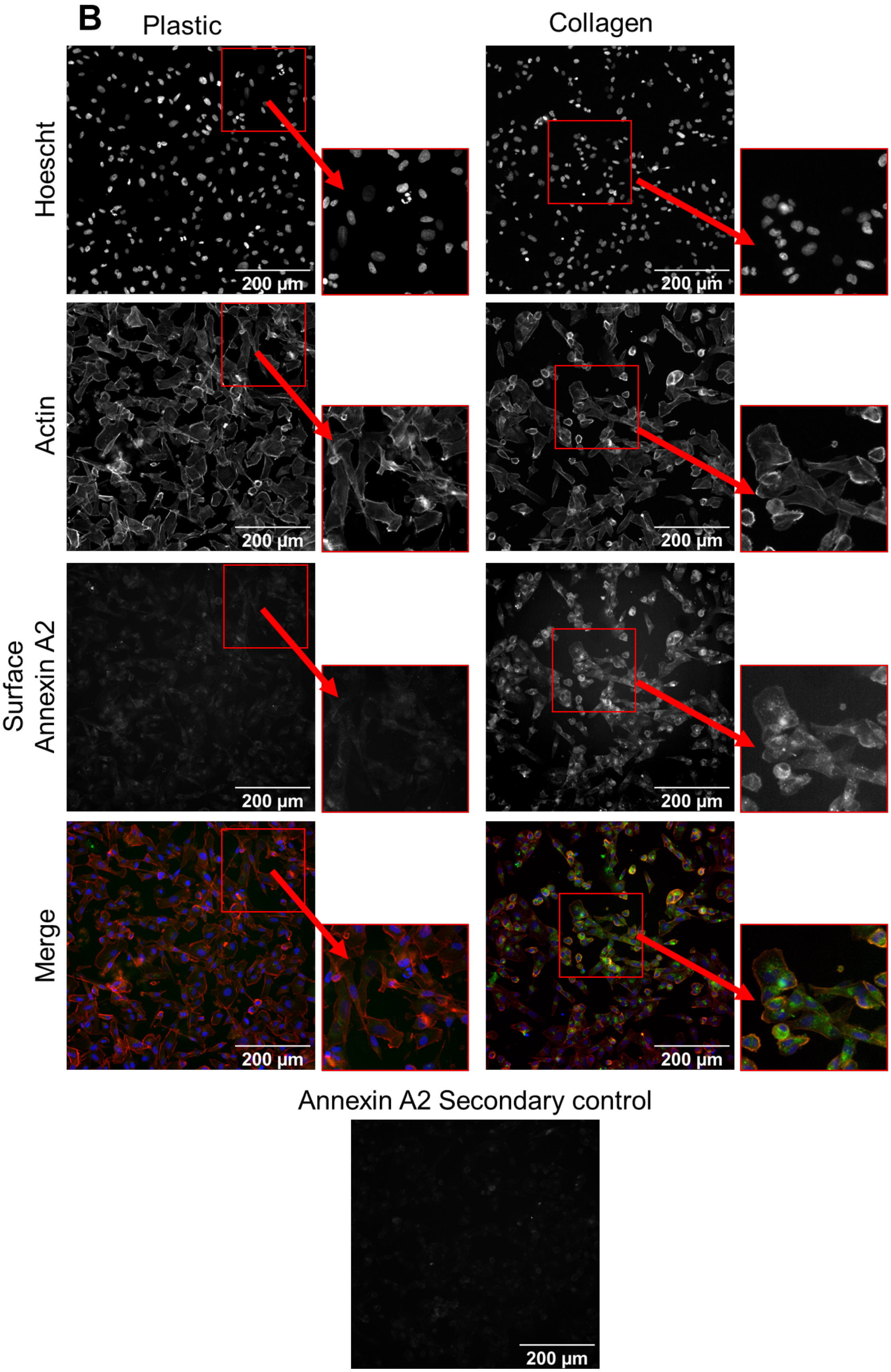
B
Plastic
Collagen
Hoescht
Actin
Surface Annexin A2
Merge
200 µm
Annexin A2 Secondary control

Supplementary Figure 2: Workflow & analysis for immunofluorescence microscopy of surface Annexin A2 staining **(A)** Images were analysed using Cell Profiler vers.3.1.9 (Broad Institute, US) (McQuin et al., 2018) according to the illustrated workflow. **(B)** Representative images show the difference in Annexin A2 staining between MDA-MB-231 cells on uncoated versus collagen coated plastics. DAPI: Hoechst staining of nuclei, TRIT-C: Phalloidin stain of actin cytoskeleton, FIT-C: Alexa-fluor-488 staining of surface Annexin A2. Secondary antibody only control shows representative image, the average fluorescence of which was subtracted from experimental data.

A Centrality statistics for FN1 in network depicted in Figure 3 A (ii)

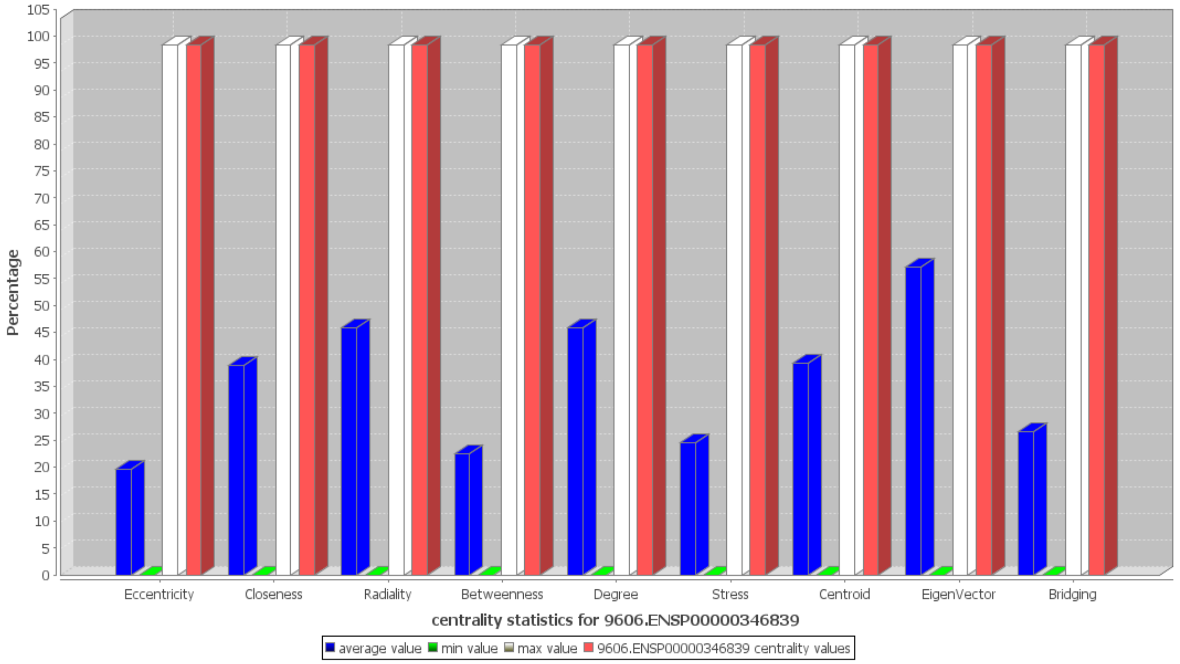

B Centiscape scatter plot (Betweeness vs Stress) for network depicted in Figure 3

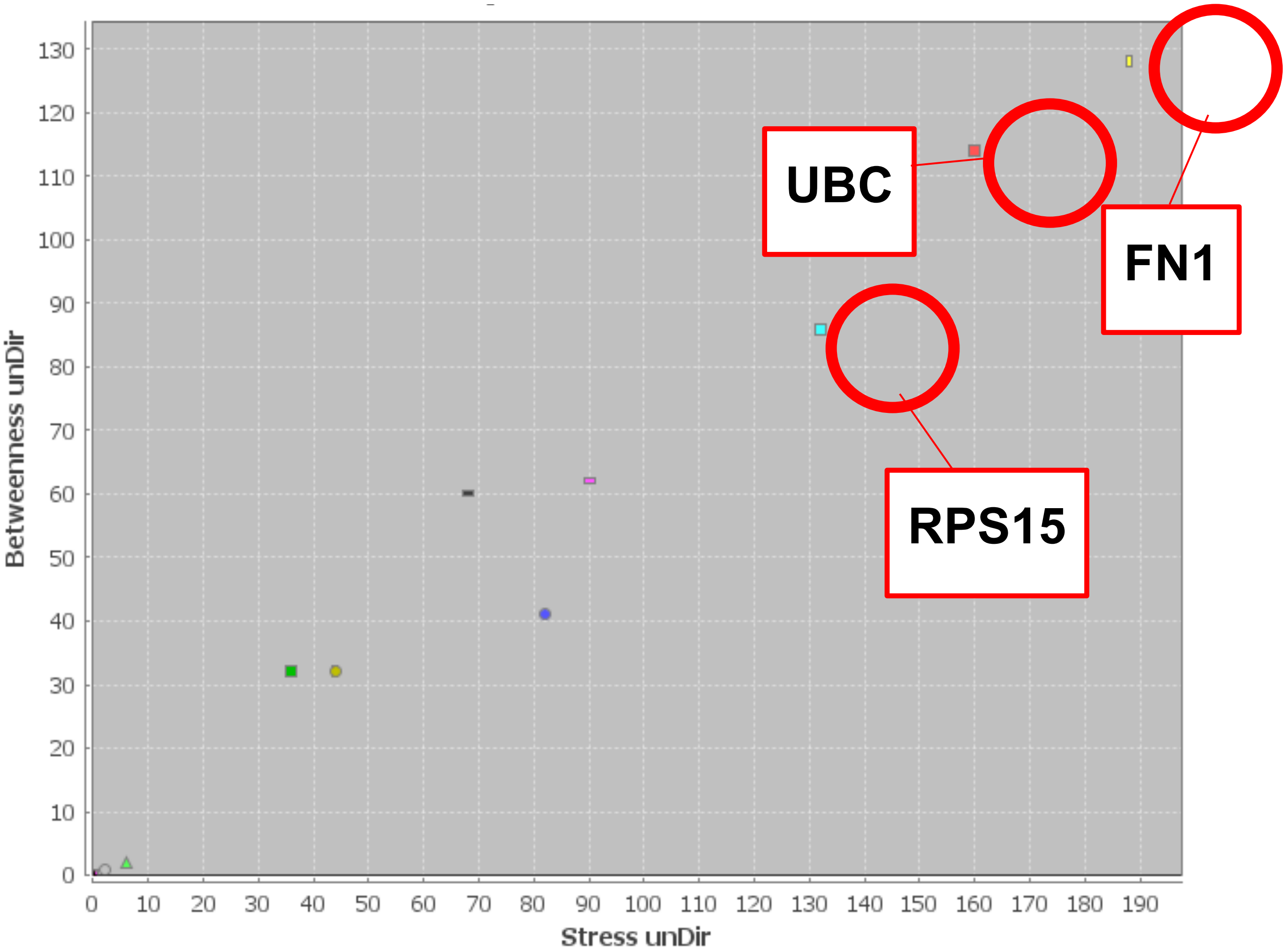

Supplementary Figure 3: (A) Centrality values for FN1 node within the Cytoscape network depicted in **Figure 3 (B)** were computed using CentiScaPe with default settings (G. Scardoni et al, 2009). For each parameter the specific node value (red), the average value (blue), the min value (green) and the max value (white) is shown. **(B)** Scatter plot of Betweeness versus Stress for network depicted in **Figure 3 (B)**, with three protein nodes of highest centrality values highlighted. The upper right quadrant would be expected to hold the most relevant nodes in a network.

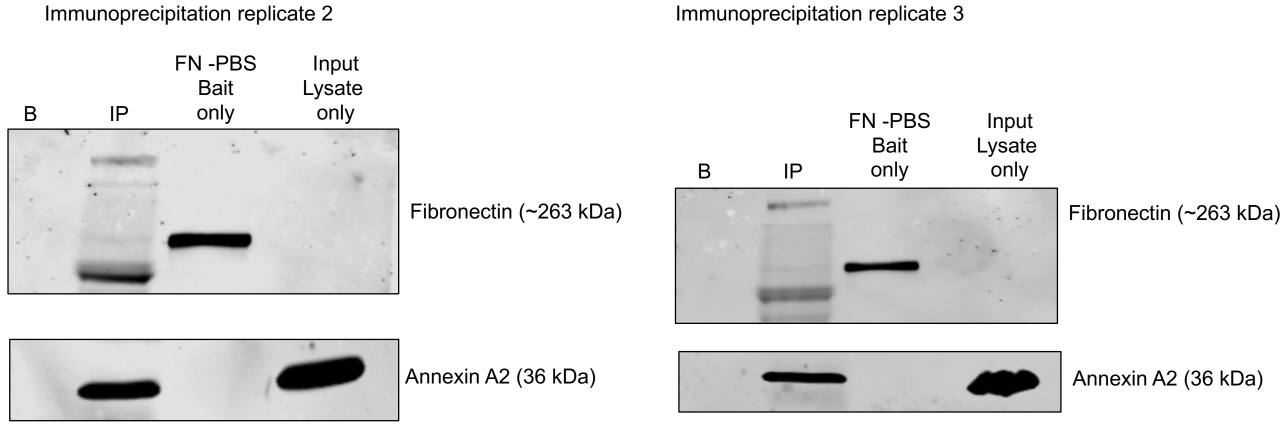

Supplementary Figure 4: Additional representative images of the Immunoprecipitation showing interaction between fibronectin and Annexin A2, with included FN-Bait only lane. B: Blank, no antibody incubation control, IP: immunoprecipitation, proteins pulled down using fibronectin baited beads from MDAMB231 cell lysate. FN-PS Bait: purified fibronectin in PBS, used to incubate with protein g agarose beads and anti-FN complex to create bead-antibody-fibronectin bait complex. Input lysate only: lysate of MDA-MB-231 cells pre-incubation with the bead-antibody-fibronectin bait complex.

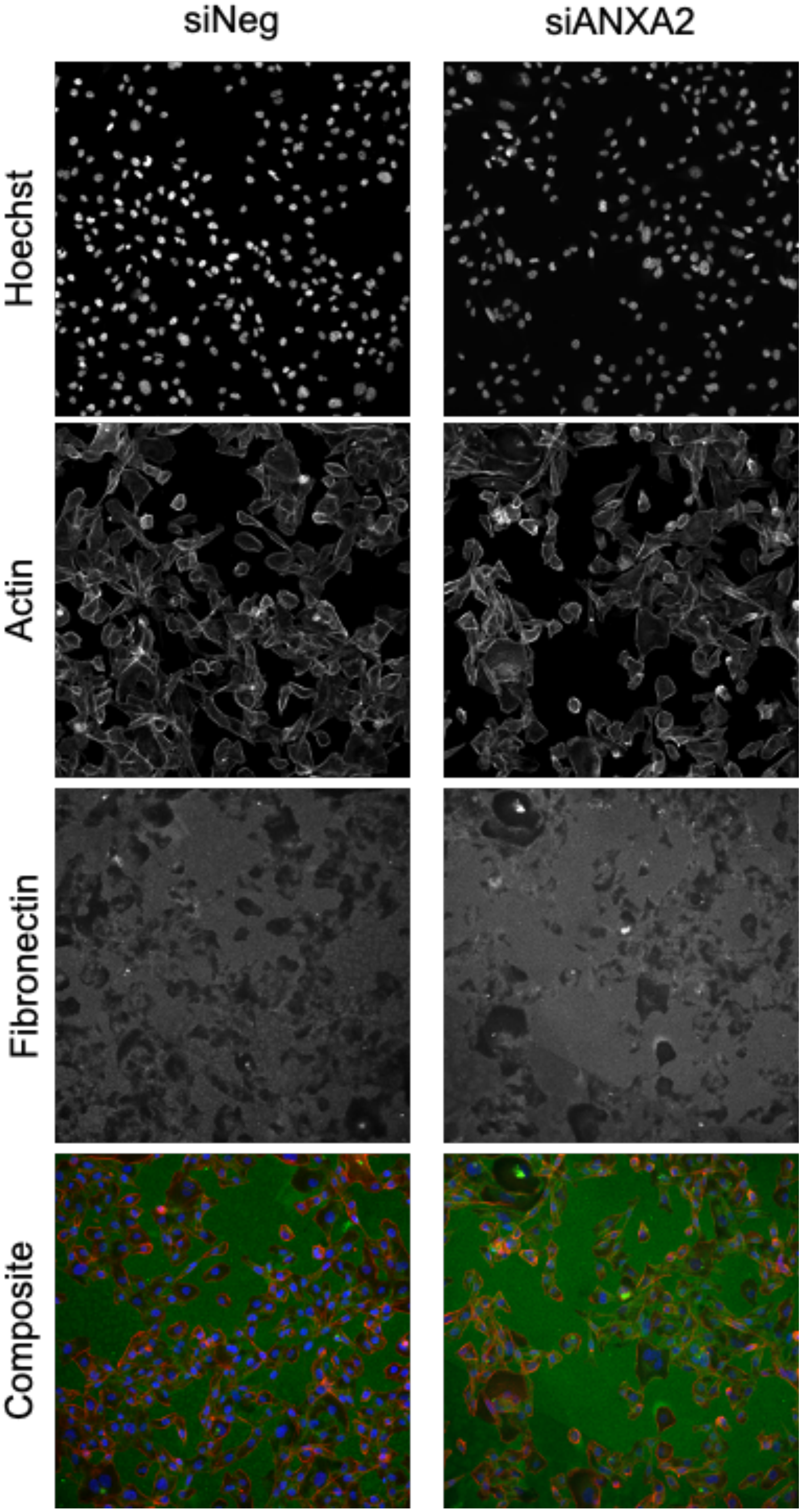
siNeg
siANXA2
Hoechst
Actin
Fibronectin
Composite

Supplementary Figure 5: Representative images of transfection negative control (siNeg) versus ANXA2 knockdown (siANXA2) MDA-MB-231 cells on fibronectin coated plastic. Showing fibronectin degradation quantification in green. Hoechst: staining of nuclei, TRIT-C: Phalloidin stain of actin cytoskeleton, FIT-C: Alexa-fluor-488 staining of fibronectin.
